# Systematically Navigating Through the Parameter Space During the Lateral Manipulation of PTCDA on the Ag(111) Surface

**DOI:** 10.1002/smtd.202500177

**Published:** 2025-05-04

**Authors:** Tim Dierker, Paul Laubrock, Philipp Rahe

**Affiliations:** ^1^ Universität Osnabrück Institut für Physik Barbarastraße 7 49076 Osnabrück Germany

**Keywords:** Ag(111), chemical bond, nanomanipulation, PTCDA, scanning probe microscopy, scanning quantum dot microscopy, tip functionalization

## Abstract

The lateral manipulation of single perylene‐3,4,9,10‐tetracarboxylic dianhydride (PTCDA) molecules serves as a key model process for both building surface‐supported nanostructures and enabling quantum sensing with single molecules attached to scanning probe microscopy tips. This work introduces an instructive procedure that guarantees the controlled lateral movement of single PTCDA molecules, in particular the isolation from molecular island edges. The lateral manipulation relies on establishing a specific bond between one of the molecular carboxylic oxygen atoms and the metallic tip of a combined scanning tunneling (STM) and atomic force microscope (AFM) before displacing the molecule by laterally moving the tip. From analyzing both the tip‐position data during this movement and the STM imaging contrast after the manipulation, a categorization scheme containing four resulting tip‐molecule‐surface configurations is proposed. Together with transitions observed between some of these configurations, the complex tip‐molecule‐surface system parameter space during the manipulation procedure can be compressed into an instructive flowchart. Following through this flowchart guarantees the lateral isolation of a single PTCDA molecule in a systematic manner and without requirement for previous knowledge. Broad applicability is verified by also manipulating molecules from Ag(111) surface step‐edges and from molecular island edges on the Au(111) surface.

## Graphical abstract

This work establishes the guaranteed controlled lateral manipulation of single perylene‐3,4,9,10‐tetracarboxylic dianhydride (PTCDA) molecules on a Ag(111) surface by utilizing a specific bond formation between a molecular atom and the scanning probe microscopy (SPM) tip. The hereby achieved expedient navigation through the tip‐molecule‐surface parameter space via an instructive flowchart serves as a fundamental basis for nanofabrication as well as tip functionalization.

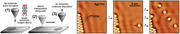

## 1 Introduction

Among the central axes of nanotechnology is the bottom‐up fabrication using single atoms or single molecules as building blocks.^[^ ^1^ ^]^ Scanning probe microscopy (SPM) emerged as a key tool for the manipulation at the atomic scale,^[^ ^2^, ^3^, ^4^, ^5^, ^6^ ^]^ with significant success in building artificial structures such as molecular cascades,^[^ ^7^ ^]^ quantum corrals,^[^ ^8^ ^]^ or covalently bonded nanostructures.^[^ ^9^ ^]^ These realizations challenge the early criticism of the “sticky finger” by demonstrating routes for atom‐by‐atom assembly.^[^ ^10^ ^]^

The manipulation process using scanning tunneling microscopy (STM), one variant of the SPM family, is nowadays well understood for many systems and often classified into the cases of *pushing*, *pulling*, and *sliding*.^[^ ^11^ ^]^ In addition, the forces required to move a single atom^[^ ^12^ ^]^ or a single molecule^[^ ^13^ ^]^ have been quantified with atomic force microscopy (AFM). Recently, machine learning approaches have been exploited for molecular manipulation.^[^ ^14^, ^15^, ^16^, ^17^ ^]^

The system of perylene‐3,4,9,10‐tetracarboxylic dianhydride (PTCDA) molecules on a Ag(111) surface is not only a representative molecule‐surface model system for which a detailed understanding of the molecule‐surface interactions has been achieved,^[^ ^18^ ^]^ but it very recently gained significant interest for preparing a probe for atomic‐scale measurements of the electrostatic potential using so‐called scanning quantum dot microscopy (SQDM)^[^ ^19^, ^20^ ^]^ as well as for sensing magnetic fields at the atomic scale.^[^ ^21^ ^]^ For these measurements, a single isolated PTCDA molecule has to be attached to the SPM tip in a highly controlled manner.^[^ ^22^, ^23^ ^]^ In contrast to earlier studies of atomic or molecular manipulation where either the local potential under the tip apex is modified to trap the species^[^ ^24^ ^]^ or where repulsive lateral molecule‐tip interaction forces are exploited to push a single molecule,^[^ ^13^ ^]^ manipulation of PTCDA molecules can be realized using a specific bond as a grip for handling the molecule.^[^ ^25^ ^]^ In particular, it has been shown that a localized chemical bond can be formed between a metallic tip and one of the carboxylic oxygen atoms.^[^ ^25^, ^26^, ^27^, ^28^ ^]^

Here, we systematically investigate the lateral manipulation procedure of PTCDA when isolating a single molecule from a PTCDA island edge. This procedure intrinsically inhibits a stochastic nature due to the restricted control of the tip structure. By classifying the experimental observations during the manipulation experiments, we introduce four categories for the resulting tip‐sample configurations and ultimately reduce the complexity of the parameter space to a flowchart diagram that systematically condenses the procedure of isolating a single PTCDA molecule on a pristine Ag(111) surface area. Thus, with this systematic approach we cannot only safely navigate through the large parameter space of the tip‐molecule‐sample configuration, but also do not require previous knowledge on the manipulation parameters. Instead, the flowchart is designed to determine suitable parameters by application to the sample system at hand.

This work is organized as follows: After introducing the experimental setup and manipulation protocol, we first describe four principal categories of tip‐molecule‐surface configurations that were identified from a large number of manipulation attempts. Physical models are proposed to explain the atomistic system behavior for each configuration. Second, we present experimental data exemplifying transitions between some of the four tip‐molecule‐surface configurations, both to strengthen the physical models and to enable the development of systematic manipulation protocols. Third, these results culminate in a manipulation flowchart that systematically incorporates the large parameter space of the PTCDA/Ag(111)‐tip system for controlled lateral manipulation. Before concluding this work, the broad applicability of this flowchart is demonstrated by applying it to the lateral manipulation of step‐edge bound PTCDA molecules and molecules on island edges on a Au(111) surface.

## 2 Experimental Section

Pristine Ag(111) surfaces were prepared under ultra‐high vacuum (UHV) conditions by Ar‐ion sputtering (energy of about 1 keV) and annealing (temperature above 500°C) cycles of a Ag bulk crystal (sourced from MaTecK GmbH, Jülich, Germany). Similar parameters were used for preparation of clean Au(111) surfaces by using a Au thin film on mica. The PTCDA molecules were evaporated in UHV from a home–build sublimator cell onto the clean sample surfaces held at room temperature. Deposition times were chosen to yield a coverage of roughly 1/3 monolayer.

Experiments were performed at low temperature (5 K) under UHV conditions using a combined scanning tunneling and atomic force microscope (type LT qPlus AFM/STM gen. III from ScientaOmicron, Taunusstein, Germany). A qPlus sensor^[^ ^29^ ^]^ with a chemically‐etched tungsten tip attached to the end of the prong was used as sensor. Before the manipulation experiments, tips were sharpened by STM‐based procedures to yield good resolution. It could safely be assumed that tips were coated by silver after this procedure. The experiments were performed in a combined STM/AFM mode where the vertical tip position (parameterized by *z*_*tip*_ that contains an unknown offset along the vertical interaction axis)^[^ ^30^ ^]^ was controlled by keeping the tunneling current at a set point *I*_*set*_ (constant‐current operation) while oscillating the tip at constant amplitude *A*_*vib*_ (zero‐peak) and measuring the AFM observables. Amplitudes of *A*_*vib*_ = 120 pm to 240 pm were typically used in this work. To resolve the molecular oxygen atom positions with strong contrast, amplitudes were increased up to *A*_*vib*_ = 720 pm where mentioned. Due to the oscillating tip, an averaged tunneling current resulting from convolving the current‐vs‐distance signal with a weighting function was measured.^[^ ^31^ ^]^ This mode is also known as dynamic STM mode.^[^ ^31^ ^]^

Lateral manipulation experiments were performed using the “Atom Manipulation” tool of the scan controller (MATRIX, ScientaOmicron) as follows: First, a set of STM parameters (*manipulation parameter set*) was defined that shall be used for the manipulation procedure during which the tip was moved laterally with an active topography feedback loop. Typical manipulation parameters were a bias voltage *V*_*gap*_ of 5 mV (bias is applied to the sample with respect to the SPM tip) and a tunneling current set point *I*_*set*_ in the range of 0.2 to 3.3 nA. Voltages in the range of −10 to 10 mV were successfully tested. STM and AFM observables were sampled at usually 600 points along the manipulation path with typical length *r* between 4 and 12 nm. Second, the raster scan was paused and the tip was parked at the designated manipulation target position. Third, the manipulation path was defined by specifying a start and end point within the STM image. To avoid movement of the tip after the manipulation, the end point of the path was chosen to be nearly identical to the parking position of the tip in step two. In step four, the movement along the specified path using the manipulation parameter set occurs when executing the manipulation procedure. Usual STM and AFM data channels were acquired during the manipulation; yet, the key information was apparent from the tip‐position *z*_*tip*_ regulated by the STM topography feedback loop. These *z*_*tip*_ data will be referred to as tip *trajectory* in the following. All image and trajectory data were herein plane‐corrected to account for the sample tilt and an offset was subtracted for convenience. A pair of arrows included in the images denotes the fast (small arrow) and slow (larger arrow) scanning directions.

## 3 Results

**Figure 1:**
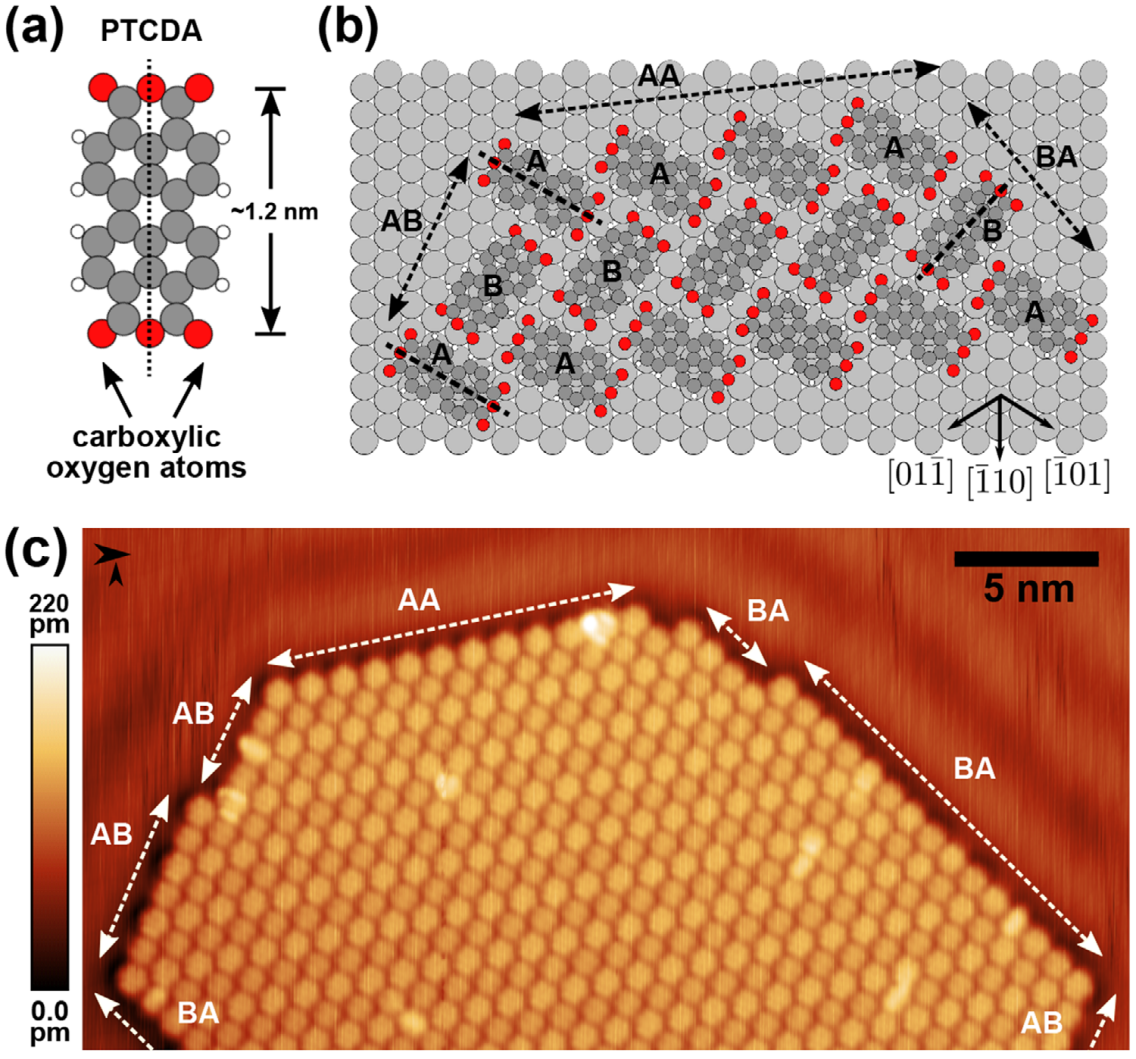
Model drawings (a) of a single PTCDA molecule and (b) of a PTCDA molecule island on the Ag(111) surface (single molecule adsorption geometries drawn according to ref. [39]). Long molecular axes marked by dashed black lines. Molecular axes of type‐A (type‐B) molecules are almost perpendicular to the step edge for the AB (BA) step type. c) STM scan of a PTCDA island showing the island edge structure (imaging parameter set: *V*_*gap*_ = −10 mV, *I*_*set*_ = 1.2 nA, and *A*_*vib*_ = 120 pm).

A PTCDA molecule consists of a perylene core terminated by two carboxylic anhydride groups on adjacent sides, see **Figure** **1**a for a sketch. The adsorption on Ag(111) surfaces (see Figure 1b) has been found to be of chemisorptive character with hybridization between the Ag 5s surface states and the lowest unoccupied molecular orbital (LUMO).^[^ ^25^, ^32^ ^]^ Geometrically, the terminating carboxylic oxygen atoms have been identified to bend towards the Ag(111) surface, leading to a non‐planar adsorption geometry.^[^ ^32^, ^33^, ^34^, ^35^ ^]^

Extended islands of PTCDA form on Ag(111) after deposition of the molecular material onto a sample held at room temperature under UHV conditions.^[^ ^36^ ^]^ In addition, decoration of the Ag(111) step edges is observed.^[^ ^37^, ^38^ ^]^ Within a molecular island, two different adsorption geometries of single PTCDA molecules on the Ag(111) surface have been identified; they are in the following referred to as type‐A and type‐B molecules. Both types of molecules adsorb with their center located above Ag surface atom bridge sites. While the long axis of a type‐A molecule is nearly perfectly aligned with the Ag(111) surface lattice, for type‐B this axis is misaligned by 17°.^[^ ^39^ ^]^ The oblique molecular unit cell with size of 1.896 × 1.261 nm^2^ is nearly rectangular with an angle of γ = 89°.^[^ ^36^ ^]^ The superstructure has been described by the matrix $\left(\begin{array}{cc} 6 & 1\\ -3 & 5 \end{array}\right)$.^[^ ^36^ ^]^ The two types of molecules are present in the form of alternating type‐A / type‐B rows.^[^ ^39^ ^]^ These two geometries can be distinguished in STM images by a slight difference in their imaged height.

Commonly, three different edge structure types of molecular islands occur as illustrated in Figure 1b,c: The first island edge structure type (**AA**) consists of type‐A molecules only, while the further two types (**AB** and **BA**) consist of alternating molecular assemblies of type‐A and type‐B molecules. The latter two types can be distinguished by the different orientation of the respective long molecular axis (dashed black line in Figure 1a) with respect to the island edge direction (see Figure 1b,c): For the **AB** (**BA**) edge structure variant, the type‐A (type‐B) long molecular axis is oriented almost perpendicular to the island edge (see Figure 1b for reference). As **BB** edges, exclusively composed of type‐B molecules, are rarely seen in our experiments, the following discussion will focus on the **AA**, **AB** and **BA** island edge structure types. Due to the Ag(111) surface symmetry, all island edge structures also exist as a version mirrored along an axis perpendicular to the island edge direction.

**Figure 2:**
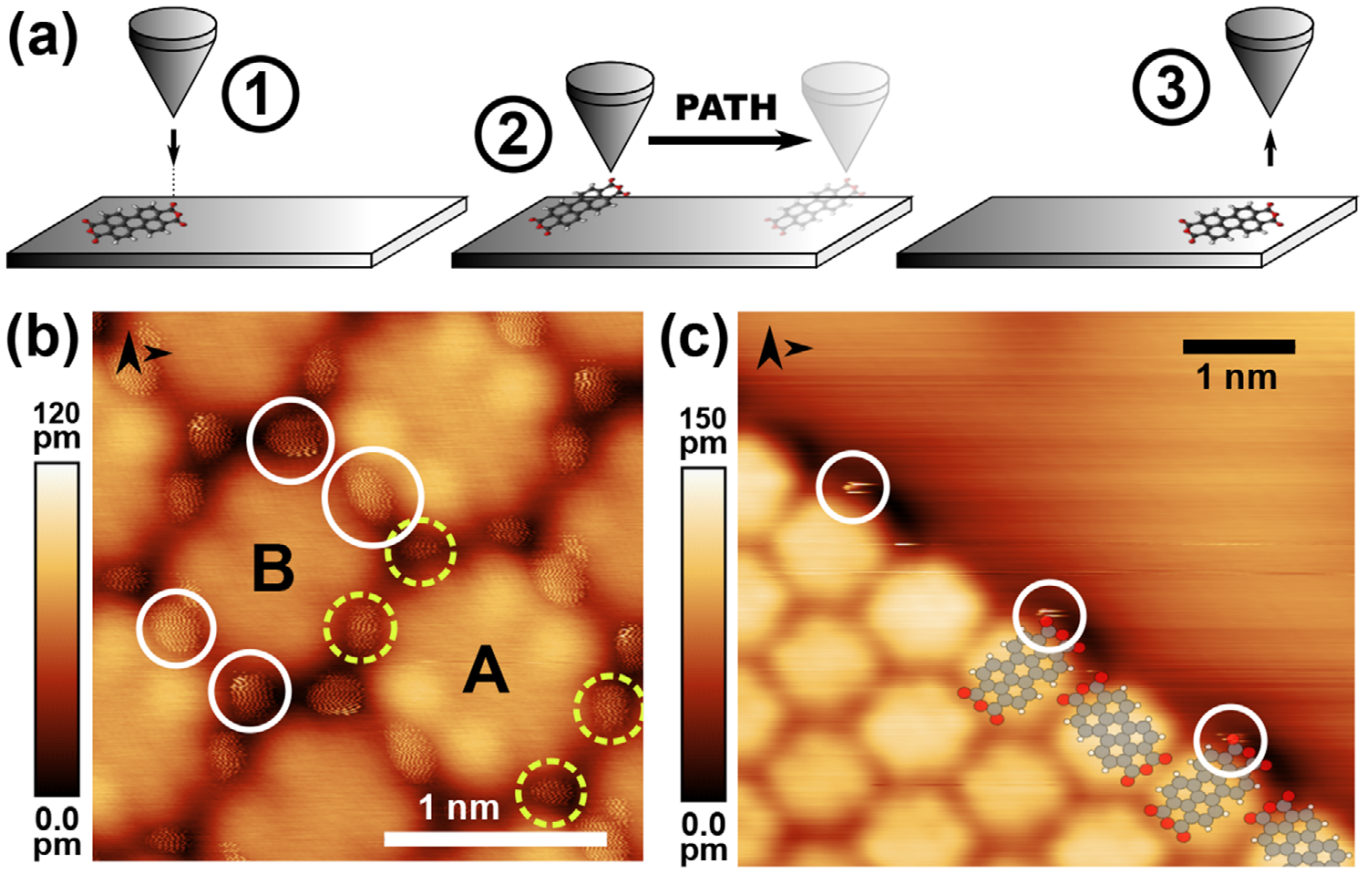
a) Illustration of the general manipulation strategy. b,c) STM topography scan of (b) a PTCDA island (*V*_*gap*_ = −10 mV, *I*_*set*_ = 1.8 nA, and *A*_*vib*_ = 720 pm) and of (c) a type‐BA PTCDA island edge (*V*_*gap*_ = −10 mV, *I*_*set*_ = 0.2 nA, and *A*_*vib*_ = 192 pm). The positions of the carboxylic oxygen atoms of type‐A (type‐B) molecules are highlighted by dashed yellow (solid white) circles.

To achieve the isolation of a single molecule away from its original island edge position, the following principle for the lateral manipulation is used as shown in **Figure** **2**a: (1) By approaching the tip closely to the position of a carboxylic oxygen atom, a bond between the metallic tip and the PTCDA molecule can form via the oxygen atom. With this tether, the molecule can (2) be dragged along the surface until (3) the bond is severed again by retracting the tip.

It is known that the carboxylic oxygen atoms of the PTCDA molecule can flip toward the Ag tip when approached closely.^[^ ^25^ ^]^ This flip can already be observed when imaging a PTCDA island or a PTCDA island edge at small tip‐sample distances (i.e. using larger tunneling current setpoints). In Figure 2b, circular features can be seen above the positions of the carboxylic oxygen atoms of the PTCDA molecules. In Figure 2c only the streaky features corresponding to the outermost carboxylic oxygen atoms are visible due to more defensive scanning parameters. These defensive parameters are chosen as otherwise molecules are uncontrollably removed from the island edge when further increasing the tunneling current setpoint in images such as Figure 2c. Thus, the first challenge of the controlled isolation of single molecules by lateral manipulation of PTCDA away from an island edge appears to be the deliberate formation of a bond between one of the outmost carboxylic oxygen atoms and the tip. Figure 1b,c shows that different island edge structures offer different options for contacting the carboxylic oxygen atom with the tip. While the outer carboxylic oxygen atoms at **AA‐**type island edges are equidistant and, therefore, equally suitable, the oxygen atoms at the other edge types express different distances towards other molecules oxygen atoms. It is recommended to choose the most exposed oxygen atoms as tip tether site for the lateral manipulation, as this avoids accidentally contacting the neighboring molecule.

### 3.1 Observation of Four Manipulation Trajectory Categories

**Figure 3:**
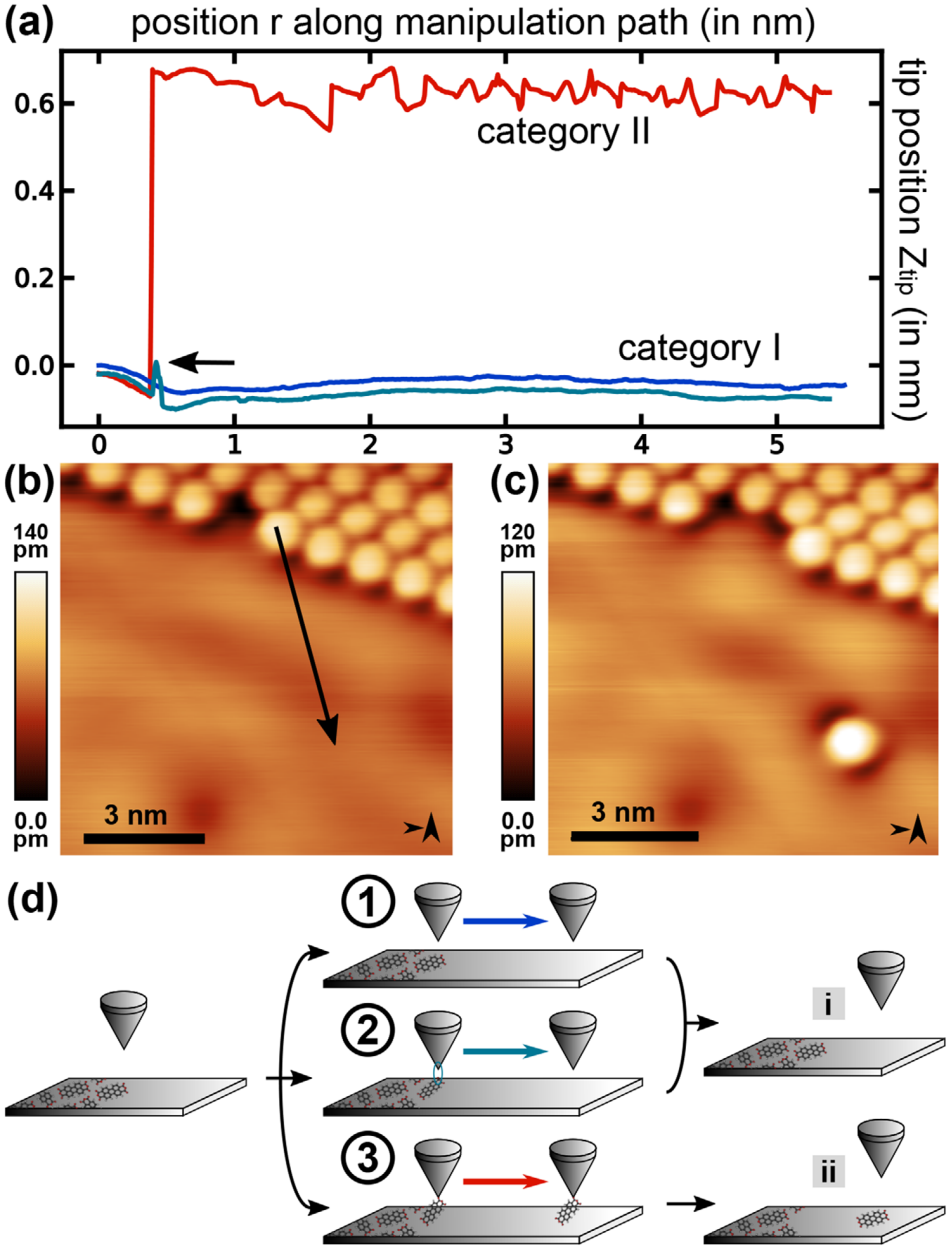
a) Trajectories along three nearly identical paths showcasing category I (blue and teal) and category II (red) (manipulation parameter set: *V*_*gap*_ = 5 mV, *I*_*set*_ = 0.2 nA (blue curve), *I*_*set*_ = 0.4 nA (teal curve), *I*_*set*_ = 0.5 nA (red curve), and *A*_*vib*_ = 120 pm). STM topography images acquired (b) before performing the lateral manipulation experiments and (c) afterwards (imaging parameter set: *V*_*gap*_ = 50 mV, *I*_*set*_ = 0.2 nA, and *A*_*vib*_ = 120 pm). The positions of the manipulation paths are marked by a black arrow in (b). d) Model drawings describing the observed trajectories that lead to tip‐molecule‐surface configurations i and ii.

**Figure 4:**
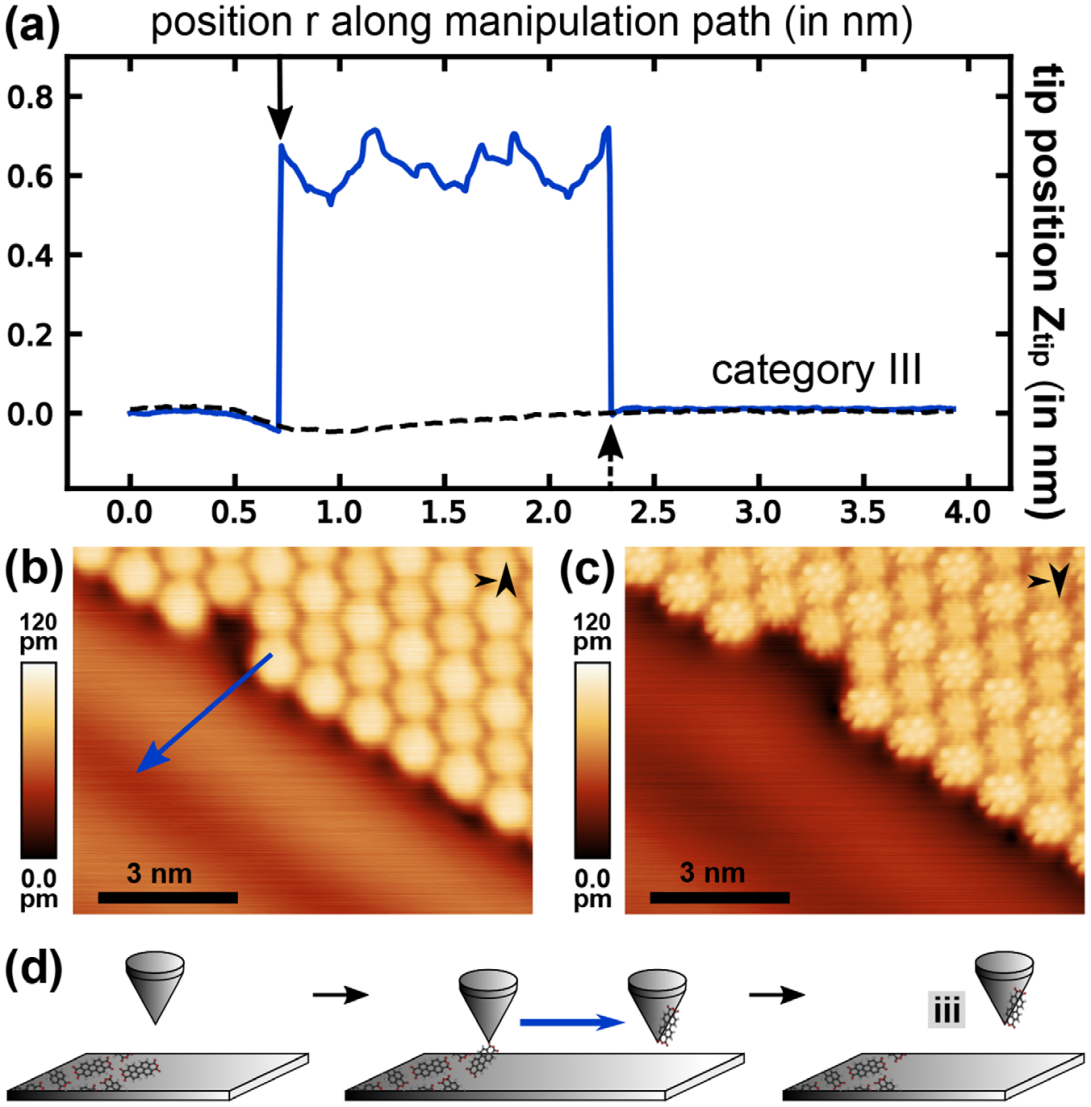
a) Representative trajectory data for a category III manipulation experiment (blue) and the trajectory data of the manipulation attempt directly before (dashed black) (manipulation parameter set: *V*_*gap*_ = −10 mV, *I*_*set*_ = 0.8 nA (blue), *I*_*set*_ = 0.6 nA (dashed black), and *A*_*vib*_ = 240 pm). b,c) STM topography images of (b) before the lateral manipulation attempt (including the location of the manipulation path marked by a blue arrow) and (c) afterwards (imaging parameter set: *V*_*gap*_ = −20 mV, *I*_*set*_ = 0.2 nA, and *A*_*vib*_ = 240 pm). d) Illustration of the physical manipulation process model leading to tip‐molecule configuration iii.

**Figure 5:**
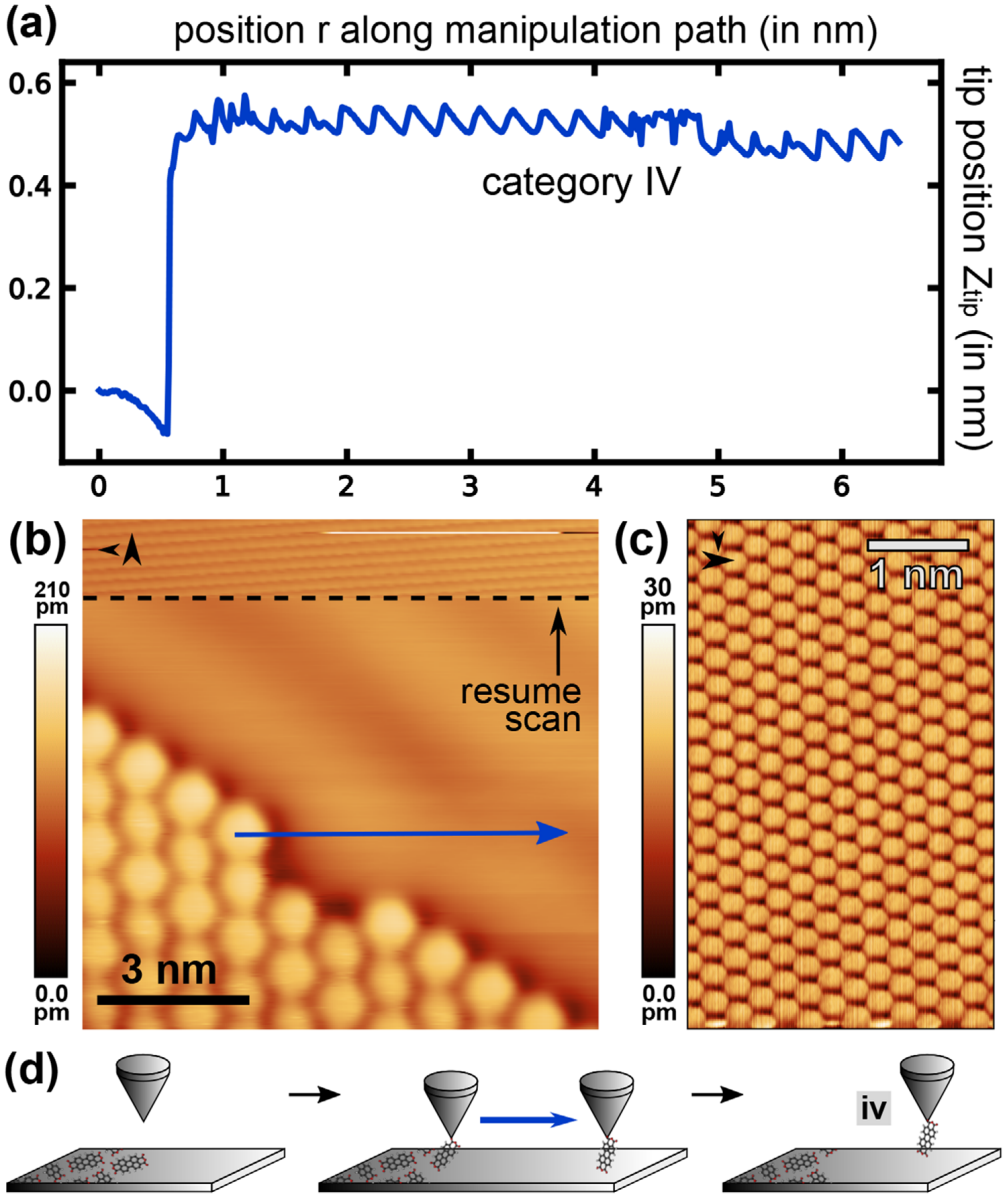
a) Representative trajectory for a category IV manipulation experiment (manipulation parameter set: *V*_*gap*_ = 5 mV, *I*_*set*_ = 0.8 nA, and *A*_*vib*_ = 120 pm). (b) STM topography image acquired step‐wise in upward direction before (below dashed black line) and after the manipulation process (above dashed black line) (imaging parameter set: *V*_*gap*_ = 20 mV, *I*_*set*_ = 0.15 nA, and *A*_*vib*_ = 120 pm). (c) Detailed view of a friction‐like contrast observed after a category IV manipulation experiment (imaging parameter set: *V*_*gap*_ = 30 mV, *I*_*set*_ = 0.2 nA, and *A*_*vib*_ = 240 pm. Image extracted from another experimental manipulation run). (d) Model of the manipulation process leading to tip molecule configuration iv.

The key data resulting from moving the tip along the manipulation path is the trajectory data, namely the tip height position *z*_*tip*_ in constant‐current feedback.^[^ ^30^ ^]^ Based on the results and observations of more than 5000 manipulation attempts at island edges, we classify the resulting trajectories into four different categories I to IV (in the following denoted in upper case roman numerals), each one leading to a respective tip‐molecule‐surface configuration i to iv (denoted in lower case roman numerals). While category I (introduced in Figure 3) describes an unsuccessful manipulation attempt, leading to configuration i whereby the molecule remains at its original position, categories II to IV denote different modifications that occur as a result of the manipulation. In particular, category II (introduced in Figure 3) represents a successful attempt with the molecule being moved to the manipulation path target location; the single isolated molecule is classified as configuration ii. Category III (introduced in Figure 4) describes an attempt during which the molecule attaches to the tip, leading to tip‐molecule‐surface configuration iii. Last, category IV (introduced in Figure 5) features a manipulation after which the molecule resides within the tip‐sample gap (denoted as configuration iv). In the following, these four categories and their respective tip‐molecule‐surface configuration will be described in detail and exemplified by experimental data.

**Figure** **3**a presents data for category I and II. A trajectory representative of category I (solid blue in Figure 3a) mostly reproduces the STM topography across a PTCDA molecule located at the island edge: The molecule is partly visible as the protrusion at the beginning of the trajectory, while the remainder maps the electronic structure of the Ag(111) surface near the island edge. This trajectory is basically identical to a line profile extracted along the manipulation path from the image data if the image was acquired with the manipulation parameter set. However, in some trajectories, a small and intermittent increase in *z*_*tip*_ is observed as a protrusion; one exemplary trajectory is included in Figure 3a (teal colored curve) and the increase in *z*_*tip*_ is marked by a black arrow. The expression of this feature is dependent on the lateral position of the manipulation path relative to the target molecule carboxylic oxygen atom position and the STM parameter set during the manipulation.

By slightly modifying the path position and the STM parameter set during manipulation, the trajectory of category I can develop into a trajectory of category II (red curve in Figure 3a). This category is distinguished by a sharp increase in the tip height (here by roughly 0.7 nm) at the position of the previous protrusion and a trajectory that remains on average at this increased tip height while expressing a corrugation pattern. After returning to the imaging STM parameter set and acquiring an image of the area after the manipulation (see Figure 3c), it is clear that a single molecule has been moved outward from the island edge (see Figure 3b for the initial situation). This molecule resides at the end position of the manipulation path.

The physical models for explaining these trajectories are depicted in Figure 3d: Starting with approaching the tip to the surface, the tip can either (1) move along the path without modifying the molecular assembly (corresponding to the blue trajectory of Figure 3a), lead to (2) an intermittent lifting of the carboxylic oxygen atom (causing the slight increase in tip height as presented by the teal trajectory in Figure 3a), or (3) form a bond to the carboxylic oxygen atom and drag the molecule along the path (as represented by the red trajectory in Figure 3a). While the molecular assembly remains unmodified in configuration i after cases (1) and (2), the molecule was translated in the third case, leading to configuration ii of an isolated molecule. Given the length of the PTCDA molecule of about 1.2 nm (oxygen–oxygen distance along long axis, see also Figure 1a), the molecule is expected to reside in the tip‐sample gap in a tilted geometry during the lateral movement along the manipulation path.

Data in **Figure** **4**a represents trajectory category III, which is defined by a similar abrupt jump at the carboxylic oxygen position after passing over the molecule (marked by the solid black arrow), but with an additional abrupt downward jump, here at about *r* ≈ 2.3 nm (marked by the dashed black arrow). This downward jump can also occur while resetting to the imaging parameter set and resuming scanning. The abrupt increase in tip height with roughly 0.7 nm is similar to a type II trajectory (red in Figure 3a). However, the STM topography images acquired before (Figure 4b) and after (Figure 4c) the manipulation experiment reveal that a molecule is indeed removed from the island edge, yet, the molecule is not present on the Ag(111) surface within the image frame. Noteworthy, the imaging contrast of the molecular island changed significantly by reproducing a complex pattern of the orbital structure.

The experimental observations are in agreement with a model where the PTCDA molecule is pulled from the island but is then transferred to the tip, likely adsorbed in a state that allows tunneling through the molecule; see Figure 4d for a sketch of this model. As a molecule‐terminated tip has a rather complex electronic structure, especially when compared to an *s*‐wave‐like metallic tip, the resulting STM topography imaging contrast emerges from a complex convolution of the surface and tip density of states^[^ ^40^ ^]^ and possibly also their higher order spatial derivatives.^[^ ^41^ ^]^ As the tip position usually returns to the original height at the second jump and convolution contrast is found from imaging the molecular island, we assume that the molecule binds to the side of the tip apex as sketched in Figure 4d. We refer to this situation (a PTCDA molecule transferred to the tip that leads to observing a *convolution contrast*) as tip‐molecule configuration iii.

Exemplary trajectory data for the fourth and final category IV are shown in **Figure** **5**a. Upon first inspection, the behavior of the *z*_*tip*_ data is similar to the case of category II: An abrupt jump at the carboxylic oxygen position after passing the island edge molecule is followed by an increased *z*_*tip*_ position along the remaining path. However, the difference to category II lies in the imaging contrast observed when resuming scanning after the manipulation experiment: As shown in the intermediate part of Figure 5b and enlarged in panel (c), the atomic lattice of the Ag(111) surface is clearly visible.

To develop the physical model for this case we first note that the trajectory data in Figure 5a expresses a periodic pattern with a periodicity agreeing with the Ag lattice constant. While similar patterns in the trajectory data are also found in some examples for category II trajectories, the difference to observing a category IV trajectory lies in the presence of atomic lattice contrast when resuming scanning on the Ag(111) surface directly after the manipulation. This *atomic lattice contrast* reminds of friction force microscopy (FFM) imaging contrast.^[^ ^42^, ^43^, ^44^ ^]^ FFM images are obtained by sliding a sharp SPM tip across a flat periodic surface such that a stick‐slip motion results, which resolves the underlying atomic lattice.^[^ ^42^ ^]^ We thus explain the category IV trajectories and the resulting atomic lattice contrast by the model depicted in Figure 5d that concludes with the tip‐molecule‐surface configuration iv: The molecule‐tip bond persists while scanning the surface and, thus, the molecule is dragged along with the tip. As the bond is strong enough to persist also at the STM scanning parameter set, a friction‐like behavior while scanning across the surface is present.

### 3.2 Transitions Between Tip‐Molecule‐Surface Configurations

**Figure 6:**
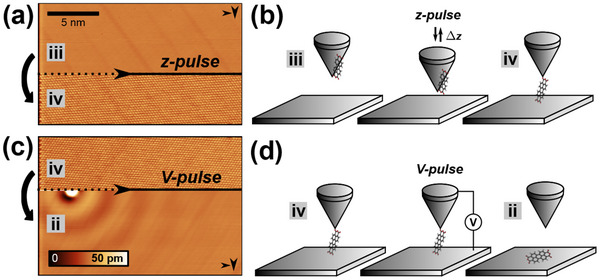
a) STM topography scan during the tip‐molecule‐surface system transition from configuration iii (upper segment) to iv (lower segment) triggered by a *z*‐pulse. Configuration iii is identified from convolution contrast when imaging PTCDA molecules (data not shown) and configuration iv from observing atomic lattice contrast (imaging parameter set: *V*_*gap*_ = 30 mV, *I*_*set*_ = 0.2 nA, and *A*_*vib*_ = 240 pm). (b) Illustration of the iii‐to‐iv transition process. (c) STM topography scan during the transition from configuration iv (upper segment) to ii (lower segment), successfully isolating a single PTCDA molecule by *V*‐pulse application (imaging parameter set identical to (a). (d) Illustration of the iv‐to‐ii transition process.

Within our extensive data set, we found category III and IV trajectories to be the most prevalent outcomes as compared to category II. Therefore, we investigate transitions between the tip‐molecule‐surface configurations ii, iii, and iv, triggered by either voltage pulse (*V*‐pulse) application or by rapidly decreasing the tip‐sample distance by an amount Δ*z* for a short duration (*z*‐pulse). **Figure** **6** shows two examples for transitions from configuration iii to iv and from iv to ii.

After transferring a PTCDA molecule to the tip as in configuration iii, indicated by a missing molecule at the island edge and by observing convolution contrast over the molecular island (e.g., after a category III manipulation attempt), temporarily reducing the tip‐sample distance by applying *z*‐pulses between 1.2 to 1.8 nm led to the observation of the atomic lattice contrast as in tip‐molecule configuration iv (see Figure 6a). As we observed a large variability of the *z*‐pulse depth within this regime, we usually start with small values and increase in 0.1 nm steps until an effect is observed. We explain this transition by movement of the molecule from the tip apex back into the tip‐sample gap. Sketches illustrating this process are shown in Figure 6b.

When observing 'atomic lattice contrast', either after a previous transition from iii to iv or after a category IV lateral manipulation attempt, performing a voltage pulse during scanning a pristine Ag(111) area (see Figure 6c) led to the molecule becoming present at the voltage pulse position. This transition therefore leads to configuration ii of a single isolated molecule. We found that voltage pulses with voltages around 0.6 V ^[^ ^23^ ^]^ for 10 to 20 ms rupture the tip‐molecule bond and deposit the molecule onto the sample surface, usually within a couple of attempts.^[^ ^45^ ^]^ As this voltage might depend on the sample system, the general strategy is to start at 0.1 V and to increase the voltage in 0.1 V steps until an effect occurs. In agreement with refs. [23, 45], the probability for molecule deposition at positive bias is observed to be higher than for negative bias. It was furthermore observed that increasing the tip‐sample distance did usually not deposit the molecule, but rather led to configuration iii where the molecule attaches to the STM tip in an undefined manner. It was also found that the molecule is occasionally deposited by accident when scanning across a molecule cluster, a molecul island edge, or a Ag step edge in close proximity while resolving the atomic lattice in configuration iv. For this reason, the image frame is chosen in a way to avoid scanning across such features when resuming imaging after the manipulation attempt.

## 4 Discussion and Development of the Manipulation Flowchart

It is known that molecular manipulation can be a complex process, involving a number of transition states, specific pathways, or a subtle balance between kinetics and thermodynamics. This complexity has very recently motivated to adapt machine learning techniques for the manipulation of single molecules.^[^ ^15^, ^17^ ^]^

**Figure 7:**
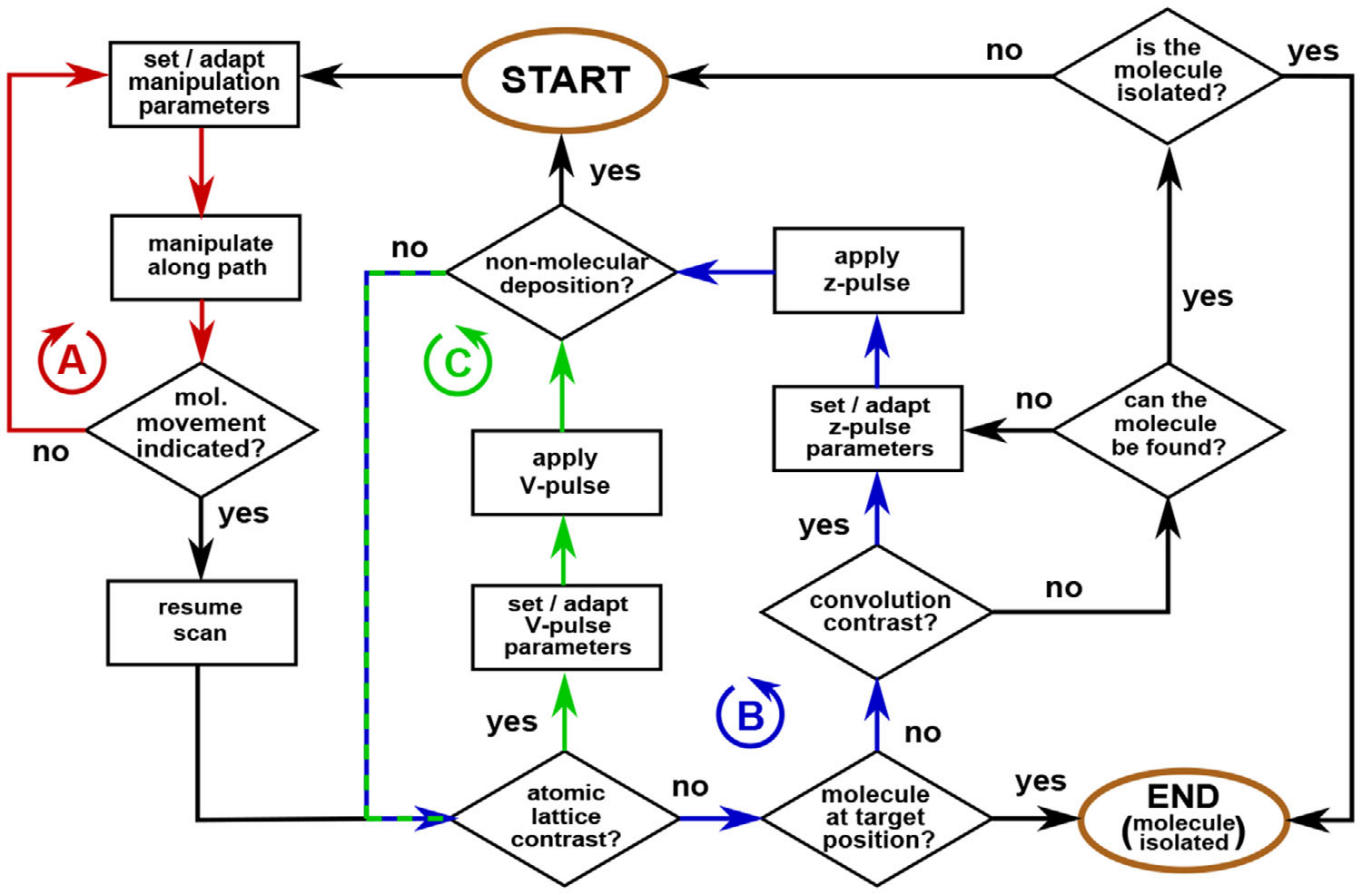
Flowchart for the lateral manipulation of single PTCDA molecules. First, the prerequisites for the manipulation experiments are met. Then, beginning at “START”, the lateral manipulation is specified and adapted until the *z*_*tip*_ data trajectory suggests molecule movement. After resuming STM imaging, checks for atomic lattice contrast and/or convolution contrast are made to identify the tip‐molecule‐surface configuration. Then, if necessary, either *V*‐pulses or *z*‐pulses are applied to trigger the respective configuration transitions. In case at any point something else than a single molecule is deposited by the tip, the lateral manipulation procedure has to be reinitialized from the start. The manipulation procedure is completed once a single molecule is successfully isolated.

From our former categorization of the experimental observations for the PTCDA/Ag(111) system, we find that the manipulation of PTCDA can be apprehended by a straightforward procedure, condensed into a comprehensive flowchart as shown in **Figure** **7**. With this procedure, it is possible to efficiently navigate through the large parameter space of the tip‐molecule‐surface system.

As prerequisites for the procedure, three requirements have to be fulfilled to begin the iteration through the flowchart at the label “START”: First, a target molecule to be isolated from a suitable island edge has to be chosen. Second, the STM tip has to be in a suitable state, ideally in a metallic *s*‐like configuration, that resolves the molecular assembly in STM images clearly. Third, the scan frame has to be positioned such that roughly half of it is covered by pristine Ag(111) surface along the slow scan axis (similar to Figure 5b). The latter requirement is important for handling tip‐molecule configuration iv: In case this configuration is found after the manipulation, a *V*‐pulse can be applied during scanning a pristine Ag(111) area in absence of obstacles such as other molecules or step edges.

The first procedure step in the flowchart is setting the initial parameters for the lateral manipulation before defining the manipulation path and attempting the manipulation along this path. In case no molecule movement is indicated from the *z*_*tip*_ trajectory data (i.e., a category I trajectory, blue data in Figure 3a), the manipulation parameter set is modified either by increasing the tunneling current setpoint *I*_*set*_ or by repositioning the manipulation path. While the former reduces the tip‐oxygen distance, the latter accommodates for the exact position of the target carboxylic oxygen atom. These three steps form “loop A” (red colored path in the flowchart of Figure 7), which is to be repeated until the trajectory data indicates molecular movement (for example by abrupt jumps or a corrugation pattern). In that case the STM imaging scan is resumed to check whether the atomic lattice is imaged at a pristine Ag(111) area. If no such atomic contrast can be observed and the single molecule resides at the target position (in this case the end point of the manipulation path), the isolation is successful via a category II trajectory (i.e., Figure 3a, red data). Should the atomic contrast not be found and should the molecule not be located at the manipulation path target position, a check for convolution contrast is made to verify the potential presence of a category III manipulation (previously shown in Figure 4). If convolution contrast is identified, applying a *z*‐pulse can trigger the transition from tip‐molecule‐surface configuration iii to iv (example previously shown in Figure 6a,b). In case the tip deposits something else than a single molecule (“non‐molecular deposition” in Figure 7), it needs to be reconditioned. Thus, a start over from the beginning is required as included by the path back to “START”. If the iii‐to‐iv transition is not successful, atomic contrast is still not achieved. If furthermore the convolution contrast persists, the previous steps are repeated and thereby form “loop B” of applying *z*‐pulses with different parameters (blue path in flowchart). If at some point during this loop or directly after a category IV manipulation (as previously shown in Figure 5) the atomic lattice contrast is found, “loop C” is entered in the flowchart (highlighted in green). This loop consists of applying *V*‐pulses with successively increasing voltage in order to trigger a safe tip‐molecule‐surface configuration transition from iv to ii. Here, it is again possible that species other than an individual PTCDA molecule are deposited and, thus, the necessity for tip‐reconditioning via a start‐over emerges. If the iv‐to‐ii transition was triggered successfully, the molecule is now isolated at the new target position (in this case the exact position of the *V* pulse application) and the flowchart is completed. Should no single molecule be found after loosing the atomic contrast, a check for convolution contrast is made to test for molecular attachment to the tip (i.e., as in configuration iii). From this situation either loop B is reentered or, if no atomic contrast is found, if the molecule does not reside at the respective target position, and if no convolution contrast is identified, one last resort is to search for the molecule within the surrounding sample area. Since we observed very rare circumstances where a deposition at sites other than the designated target position occurred during our experiments (for example at the scan frame edge, at other molecule clusters, at step edges, or at island edges in close proximity) the molecule could be isolated by chance. If no molecule can be found in the near vicinity of the manipulation area, there is the possibility it transferred to the tip and can be recovered by applying *z*‐pulses. However, we note that this path often leads to depositions of non‐single molecular nature. Still, the flowchart would be restarted via this route and, thus, ensures consistency.

The extensive data set acquired during the process development suggests that the most probable path through the flowchart process for isolating a single molecule starts with iterations of loop A until a category IV manipulation trajectory is observed, then proceeds via verifying atomic lattice contrast (tip‐molecule‐surface configuration iv), followed by voltage pulse application through loop C (to trigger the iv‐to‐ii transition) until finally an isolated molecule is present.

## 5 Transferability of the Manipulation Protocol

In order to highlight the broad applicability of the flowchart approach, we finally present two successful examples of manipulating molecules embedded into Ag step edges in Section 5.1 as well as of manipulating molecules at island edges on the Au(111) surface in Section 5.2.

### 5.1 Transfer to Ag Step Edge Bound Molecules

**Figure 8:**
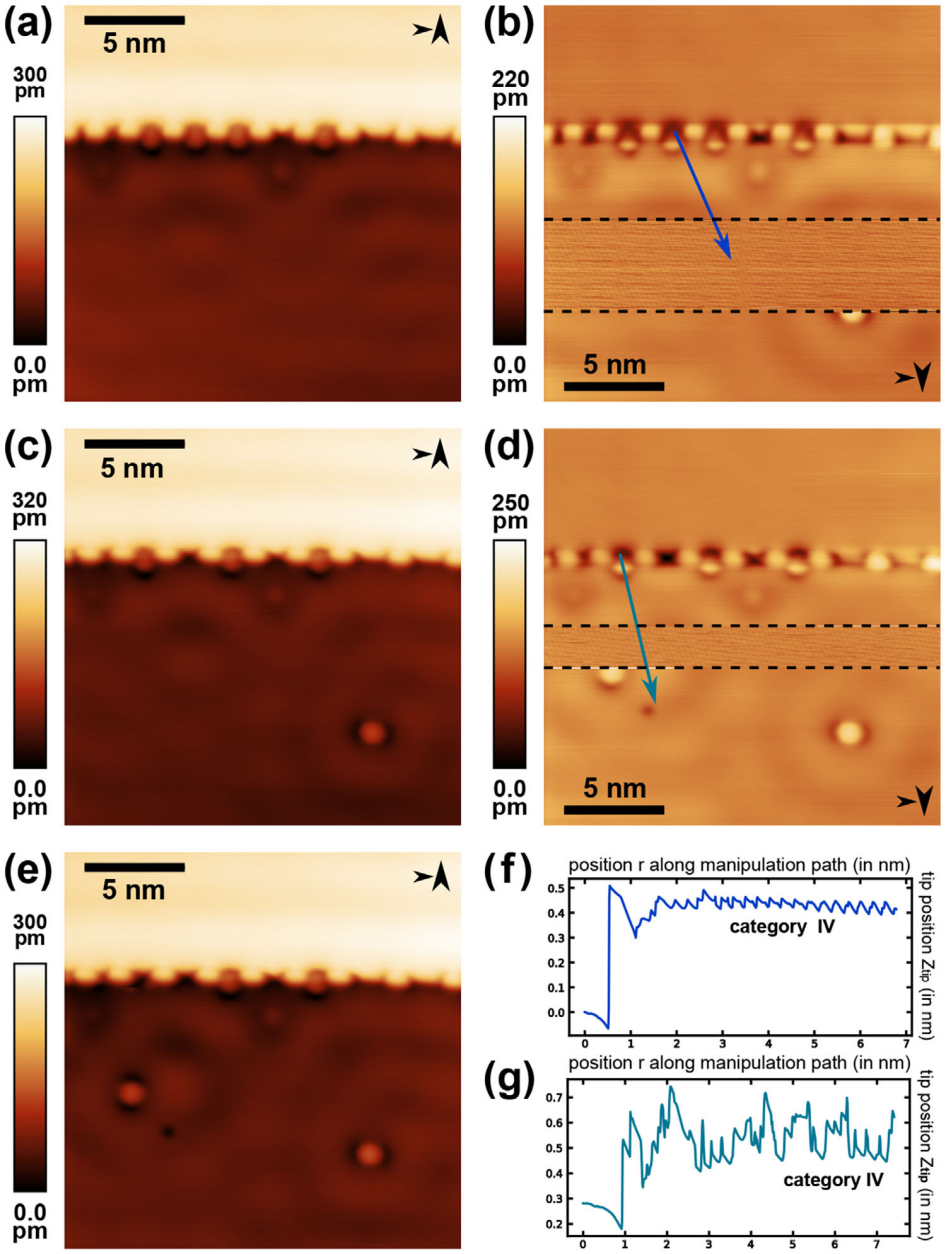
Two representative examples leading to isolated PTCDA molecules after applying the lateral manipulation flowchart to Ag step edge embedded molecules. a,c,e) STM images before and after the respective manipulation attempt (b,d) Line‐by‐line corrected, downwards scanned STM images, paused for the lateral manipulation attempt. Both images express atomic lattice contrast across the scanned area between the black dashed lines. Specified manipulation paths are included as (b) blue and (d) teal colored arrows (imaging parameter set: *V*_*gap*_ = 20 mV, *I*_*set*_ = 0.1 nA, and *A*_*vib*_ = 120 pm). f,g) *z*_*tip*_ trajectory data of the lateral manipulation corresponding to the (f) blue and (g) teal colored paths in the images (b) and (d) (manipulation parameter set: *V*_*gap*_ = 5 mV, *I*_*set*_ = 1 nA, and *A*_*vib*_ = 120 pm).

The flowchart manipulation process is applied to molecules attached to Ag step edges. Thereby, we transfer the procedure to a different PTCDA binding configuration of the starting geometry. The same three prerequisites are met prior to starting the flowchart procedure; yet, a step edge decorated with PTCDA molecules is chosen instead of a molecular island edge. A series of consecutive STM images is presented in **Figure** **8** with the slow scanning direction alternating between up and down. Imaging is paused during the downward scanned images in Figure 8b,d to attempt two molecular isolations of step‐edge embedded molecules by specifying the manipulation paths marked by the colored arrows. After several iterations through loop A in the flowchart, for each attempt a trajectory of category IV was observed. The respective trajectory data is reproduced in Figure 8f,g. Atomic lattice contrast is found when resuming scanning, thereby indicating tip‐molecule‐surface configuration iv. Following the flowchart, loop C is thus entered and voltage pulses are applied until the transition from configuration iv to ii is triggered by a molecule deposition. The images in Figure 8c,e each confirm the successful isolation by displaying the single molecules in the lower half of the images at the respective positions of the voltage pulse applications. As a proof‐of‐concept, a total number of over 80 molecules were isolated from step edges in a similar manner following the procedure provided by the flowchart (data not shown).

### 5.2 Transfer to Molecular Island Edges on the Au(111) Surface

**Figure 9:**
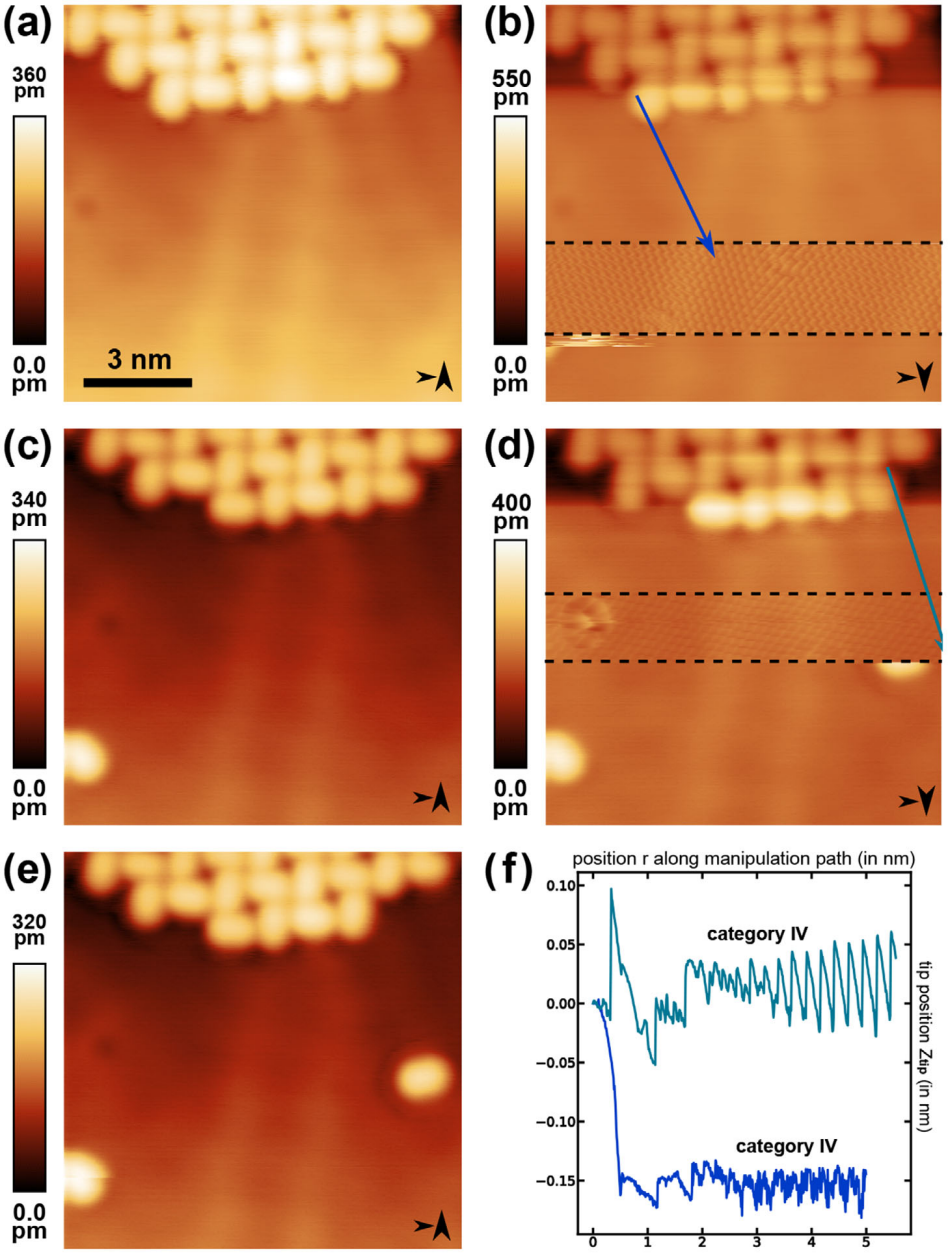
Two representative examples leading to isolated PTCDA molecules after applying the lateral manipulation flowchart to molecules on island edges on the Au(111) surface. (a,c,e) STM images before and after the respective manipulation attempt. (b,d) Line‐by‐line corrected, downward scanned STM images, paused for the lateral manipulation attempt. Both images express atomic lattice contrast within the area between the black dashed lines). Specified manipulation paths are included as (b) blue and (d) teal colored arrows (imaging parameter set: *V*_*gap*_ = 20 mV, *I*_*set*_ = 5 pA and *A*_*vib*_ = 120 pm). (f) *z*_*tip*_ trajectory data of the lateral manipulation corresponding to the blue and teal colored paths in the images (b) and (d) (manipulation parameter set: *V*_*gap*_ = 5 mV, *I*_*set*_ = 2.5 nA (blue), *I*_*set*_ = 2.0 nA (teal) and *A*_*vib*_ = 120 pm).

In addition, the flowchart method is applied to PTCDA molecules at molecular island edges on the Au(111) coinage metal surface. It is ensured that the prerequisites for the lateral molecule isolation are met before attempting the manipulation with a Au‐terminated tip. During the experiments on the Au(111) surface, all four trajectory types, as well as four tip‐molecule configurations could be found (data not shown). Examples of two successive isolations are shown in **Figure** **9**. Two lateral molecule manipulations along the paths marked by the colored arrows in Figure 9b,d are performed with their respective *z*_*tip*_ trajectory data reproduced in Figure 9f. Both trajectories are identified as category IV as atomic lattice contrast is found after resuming the scanning process (see Figure 9b,d). The molecular isolation was finally achieved by triggering the iv‐to‐ii transitions via *V*‐pulse application.

Interestingly, the imaging and manipulation parameter sets leading to successful molecular isolation differ slightly from the ones used on Ag(111). In particular, a smaller current setpoint was chosen for imaging as isolated molecules tended to be dragged along during scanning otherwise. This may possibly correspond to the PTCDA molecules being more weakly bound to the Au(111) surface when compared to their binding to the Ag(111) substrate.^[^ ^18^, ^35^ ^]^ Along the same line, contacting the carboxylic oxygen did not result in an increase in tip height as featured prominently in Ag(111) trajectories. We speculate that this might as well be caused by the weaker bond between the molecule carboxylic oxygen atoms and the underlying substrate, which results in a more planar geometry with oxygen atoms at larger *z* position.^[^ ^18^, ^35^ ^]^ However, it was required to choose larger current setpoints for the manipulation parameter set for removing the target molecules from the island edge on Au(111), which we tentatively explain by the weaker bond between the Au‐terminated tip and the molecule. It is, however, possible to determine molecule movement from the corrugation featured in category II to IV tip‐height trajectories. Overall, the flowchart could directly be followed for the PTCDA/Au(111) system and parameters for successful molecular manipulations were identified in a straightforward manner.

## 6 Conclusion

In conclusion, we systematically investigated lateral manipulation of PTCDA molecules on a Ag(111) surface. By forming a specific bond between the tip and a carboxylic oxygen atom of an island edge molecule and then moving the tip laterally away from this island edge, single molecules can be translated in a most controlled manner. Based on the data from many experimental runs, we classified the manipulation attempts into four different categories, each leading to a respective tip‐molecule‐surface configuration. Together with the possibility to trigger transitions between some of these configurations, we compressed the complex parameter space of the tip‐molecule‐surface system during the manipulation into an instructive flowchart. Following through this flowchart guarantees the isolation of a single PTCDA molecule away from an island edge. Furthermore, we successfully transferred the flowchart approach to Ag step‐edge bound molecules and molecules at island edges on the Au(111) surface to prove its broad applicability. Systematic navigation through the parameter space is expected to significantly improve the efficiency for both nanofabrication and tip functionalization.

## Conflict of Interest

The authors declare no conflict of interest.

## Data Availability

The data that support the findings of this study are available from the corresponding author upon reasonable request.
